# Supplementary material for: Predation and fragmentation portrayed in the statistical structure of prey time series
Source: BMC Ecol. 2009 May 6;9:10. doi: 10.1186/1472-6785-9-10 (PMC2689204; doi:10.1186/1472-6785-9-10)
Supplement: Additional file 2 — Voles and related classes ODDox Documentation. ODDox documentation of the agent-based model (ALMaSS) applied by Hendrichsen et al. The documentation is started by activating main.html. [file 1472-6785-9-10-S2.zip › Vole_ODDox/setaside_8cpp.html]

ALMaSS ODDox: setaside.cpp File Reference

- Main Page
- Related Pages
- Classes
- Files

# setaside.cpp File Reference

---

## Detailed Description

**Setaside.cpp This file contains the source for the setaside class**   

by Chris J. Topping   
Version of June 2003   
All rights reserved.   
  
With additions as noted in:   
Doxygen formatted comments in July 2008   

`#include "ls.h"`  
`#include "setaside.h"`  

|  |  |
| --- | --- |
|  |  |
| Functions | |
| CfgInt | cfg\_setaside\_glyphosate ("CFG\_SETASIDE\_GLYPHOSATE", CFG\_CUSTOM,-9999) |

---

## Function Documentation

|  |  |  |  |  |
| --- | --- | --- | --- | --- |
| CfgInt cfg\_setaside\_glyphosate | ( | "CFG\_SETASIDE\_GLYPHOSATE" | , |  |
|  |  | CFG\_CUSTOM | , |  |
|  |  | - | *9999* |  |
|  | ) |  |  |  |

Referenced by SetAside::Do().

---

Generated on Thu Jan 22 14:13:45 2009 for ALMaSS ODDox by 
 1.5.6
